# A Plea for a Sub-Specialty in Dentistry

**Published:** 1902-04

**Authors:** C. M. Wright

**Affiliations:** Cincinnati, Ohio


### A PLEA FOR A SUB-SPECIALTY IN DENTISTRY.[1]

BY C. M. WRIGHT, D.D.S., CINCINNATI, OHIO.

In 1864 I read an essay before the Cincinnati Dental Society—the mother of our present Odontological Society of Cincinnati—on "Specialties in Dental Practice." This was my first paper prepared for a dental meeting. At that time I could conceive of but two departments, the Mechanical and the Operative, and I offered arguments in favor of a division with all the optimistic assurance of youth.

About twenty-five years ago, in Basel, Switzerland, I mapped out a scheme for the practice of a new specialty of dentistry for a woman of education who applied to me for advice. She wished to earn a living, yet did not desire, or feel able, to enter into the full work of an accomplished Doctor of Dental Surgery. I then planned for her the kind of work which shall form the subject of my talk this evening. She did not follow my suggestions and fit herself for the specialty, because it was not feasible at that time and place, but this circumstance did not affect my opinion of the excellence of the idea.

The time has arrived when I believe we should make it possible for and encourage just such applicants to enlist in this field of useful service.

Ten years ago I explained the same scheme to another lady who sought advice about entering the profession of dentistry. This lady was convinced by my picturesque and enthusiastic advocacy of the "Specialty within a Specialty," but as there appeared no opportunity for acquiring the education necessary for the practice of the vocation, she was compelled to abandon the plan. Soon after that she was enrolled as a student in a school of stenography, and now spends ten hours a day agitating a type-writer in a down-town business office.

The recent papers by Dr. D. D. Smith, of Philadelphia, on the prophylactic value of a certain dental operation,—viz., *the expert polishing of the human teeth,* beginning with the children, and having regular and frequent appointments and systematic attention in this one direction, and continuing it possibly throughout

---

[1] Read before the Odontological Society of Cincinnati, January 31, 1902.

life,—has appealed to me so forcibly that I have felt that suggestions on "A Sub-Specialty in Dentistry," devoted to the polishing of teeth and the massage of gums, might be apropos. I beg leave, then, to offer the following:

1. The practitioners of this separate and yet most important branch of dentistry are to be women,—women of education and refinement,—who are seeking a field for work of an honorable and useful kind and among people of culture.

2. The dental colleges are to offer opportunities for this partial and separate training. The course to consist of lectures on the Anatomy of the Teeth and Gums, Special Pathology, and Physiology, and a special clinical training in prophylactic therapeutics.

3. Upon the completion of this special course, which shall require one session, or one year of study, and practice under instruction in the college infirmary, and after presenting satisfactory evidence of proficiency in the polishing of teeth and caring for the mouth, the college shall grant a certificate of competence to the graduate of this course.

4. With this training and the dental college certificate, these ladies may be employed by dentists for this special work, or may practise the same at parlors of their own, or at the homes of patients, the dentists using their influence and recommending the new specialists, just as physicians and surgeons recommend and insist upon the services of the trained nurse or the masseuse.

This is but an outline of a scheme, the details of which seem easy of arrangement.

I think every one of you will agree with me that there could be no more valuable service in oral hygiene than just such a class of specialists would afford.

Dentists who treat pulps, fill teeth, make bridges, crowns, and plates, treat inflammation and Riggs's disease, extract teeth and roots, and construct and care for regulating appliances do not devote the proper attention to the careful polishing of all the surfaces of the thirty-two teeth, nor to the frequent massaging of the tissues which Drs. Smith, Talbot, and others advocate so strenuously as essential to the health of the human mouth.

We, as a profession, have neglected these operations. We scale off the calcareous and other accretions at long intervals, often imperfectly, or partially and hurriedly, and with wheels and brushes on our electric engines whisk off the most conspicuous stains, leaving the teeth only comparatively presentable. We seldom perform the operation with satisfaction even to ourselves. Probably, in the light of the revelations made by Dr. Smith, the majority of us have never once in our lives thoroughly polished all the exposed surfaces of the teeth of our patients.

I think every one would be glad to have this work done by experts, fortnightly or monthly, for every patient who comes to us for so-called dental operations,—viz., for crowns, fillings, bridges, etc.,—and not only before coming, but at regular intervals afterwards, *especially in conjunction with our surgical treatment of Riggs's disease.*

I have claimed that teeth are a luxury, but *clean* teeth—and by this I mean teeth each one of which has been polished on every surface by a skilled operator until it presents a finish only rivalled by some fine jewel—should be a badge of refinement that would place the child, the man, and the woman on a certain social plane.

Polished teeth in this age of luxury, when the bath, the manicure, the chiropodist are considered necessities, should form a subtle reason for an aristocracy of cleanliness which is next to godliness. Our ideas of the term *clean* have changed during the last twenty years. Then a man was clean who took a Saturday-night bath, a monthly shampoo, and shaved himself three times a week. Now we talk about "surgical cleanliness," and know about infections on toothpicks, and even upon smooth-looking enamel.

As we advance in the adoption of luxuries, we get more particulars about daintiness, and this seems to be true in all things excepting with the teeth. I believe that it is largely due to us that this surgical cleanliness has not taken a more prominent place in the estimation of the general public. Our devotion to the diversified and exhausting mechanical operations which we are hourly called upon to perform, and on account of which we have gained reputation as a skilled and useful profession, has diverted our thoughts from what we call a minor operation.

Our energies, measuring as much per foot-pound as that of any other profession,—law, medicine, or theology,—have been fully expended on the many more brilliant operations in our surgical repertory, and we have neglected this one, which we all admit is as important as any in its relation to health. We have given ourselves over to restoration and been content to advise tooth-brushes, sanatol, or vegetol to our patients, leaving the responsibility of real prophylaxis with them. We may not be able to change our modes and habits of practice, but we can, by this method and with the hearty co-operation of the dental colleges in affording the educational equipment necessary for the cultivation of this field of special practice, revolutionize dentistry—place it upon a still higher plane. The operation suggested is more directly in the line of preventive medicine, with all that this implies, than any other in the scope of prophylaxis that I can think of, such as boiled drinking-water, ventilation, sanitary plumbing, physical exercise, diet, and bathing. Imagine a room full of children, as they are now in any school, public or private, in regard to surgically clean mouths, and the same children after a thorough polishing of all their teeth. Here is an opportunity for missionary work. Enthusiasm on the part of operator and patient could easily be stimulated, and health and morals be vastly improved. Ten years of such effort on the part of our profession would do more for the human family than all the tooth-pastes and powders ever invented, or all the tracts for the people ever published, for the responsibility would be removed from the patient and placed where it belongs,—on the practitioner of this art of oral hygiene, these sub-specialists.

We have set the men on pedestals who have been able to cut out a carious spot on a tooth, extend and form a cavity so that a clean surface of gold may take the place of enamel and protect one part of a single tooth from a single disease; shall we not commend and honor the specialist who patiently and regularly operates for the prevention of this and other diseases by intelligent and systematic care of the entire mouth? This is a fundamental idea of dentistry, agreed to by all and yet neglected.

With our present exact knowledge of etiology and our increasing familiarity with the wide-reaching effects of oral sepsis, are we not ready for the establishment and hearty endorsement of trained specialists who will devote their entire time to this one branch of prevention? From personal observation among refined people in America and Europe, I believe success would follow the efforts of the colleges and the profession in this direction, for we shall be supplying an awakening demand for just such service.

